# Children as ethnobotanists: methods and local impact of a participatory research project with children on wild plant gathering in the Grosses Walsertal Biosphere Reserve, Austria

**DOI:** 10.1186/s13002-016-0119-6

**Published:** 2016-10-10

**Authors:** Susanne Grasser, Christoph Schunko, Christian R. Vogl

**Affiliations:** Working Group Knowledge Systems and Innovation, Division of Organic Farming, Department of Sustainable Agricultural Systems, University of Natural Resources and Life Sciences Vienna (BOKU), Gregor-Mendel-Strasse 33, 1180 Vienna, Austria

**Keywords:** Participatory research, Participatory video, Citizen science, Ethnobotany, Ethnobiology, Research with children, Wild gathered plant species, Local knowledge

## Abstract

**Background:**

Ethically sound research in applied ethnobiology should benefit local communities by giving them full access to research processes and results. Participatory research may ensure such access, but there has been little discussion on methodological details of participatory approaches in ethnobiological research. This paper presents and discusses the research processes and methods developed in the course of a three-year research project on wild plant gathering, the involvement of children as co-researchers and the project’s indications for local impact.

**Method:**

Research was conducted in the Grosses Walsertal Biosphere Reserve, Austria, between 2008 and 2010 in four research phases. In phase 1, 36 freelist interviews with local people and participant observation was conducted. In phase 2 school workshops were held in 14 primary school classes and their 189 children interviewed 506 family members with structured questionnaires. In phase 3, 27 children and two researchers co-produced participatory videos. In phase 4 indications for the impact of the project were investigated with questionnaires from ten children and with participant observation.

**Results:**

Children participated in various ways in the research process and the scientific output and local impact of the project was linked to the phases, degrees and methods of children’s involvement. Children were increasingly involved in the project, from non-participation to decision-making. Scientific output was generated from participatory and non-participatory activities whereas local impact - on personal, familial, communal and institutional levels - was mainly generated through the participatory involvement of children as interviewers and as co-producers of videos. Creating scientific outputs from participatory video is little developed in ethnobiology, whereas bearing potential.

**Conclusions:**

As ethnobotanists and ethnobiologists, if we are truly concerned about the impact and benefits of our research processes and results to local communities, the details of the research processes need to be deliberately planned and evaluated and then reported and discussed in academic publications.

## Background

Ethically sound research in applied ethnobiology should benefit local communities by giving them the possibility “to actively participate in all phases of research and related activities from inception to completion, as well as in application of research results” (Principle 5 of the ISE Code of Ethics) [1]. Such approaches to research were summarized under *participatory research*, roughly defined as research *with* rather than *on* people [2], whereas the degrees of involvement of local communities can range from punctual involvement, as in citizen science [3], to full involvement, as in co-enquiry [4]. The benefits for local communities generally include assistance in solving problems as perceived by themselves [2], but in detail depend on the degree as well as the quality of local people’s involvement [5]. In citizen science projects benefits were suggested to include enhancements in environmental democracy, scientific literacy, social capital, citizen inclusion in local issues, benefits to government and benefits to ecosystems [6].

In ethnobiology a diversity of local actors have participated in research, whereas the active involvement of children, common in diverse fields of research [7], has not yet been seen in ethnobiological projects (one exception is a Bulgarian study in which high school pupils were asked to interview family members about their wild plant uses [8]). However, children are increasingly being addressed as research objects in ethnobiological research, for example in Mexico [9], El Salvador [10], Venezuela [11], Bolivia [12], the USA [13], Benin [14], India [15] and Thailand [16].

The meaning of *participation* is contested in the body of literature on participatory research with children. It is argued that much of what is called participatory research with children is in fact research that is adult-designed, adult-led and adult-managed rather than adult-supported [17]. It has also been shown that only in a very few cases does research including the terms community-based participatory research and youth adopt a partnership approach to research with children in some phases of the research project [18]. Genuine participation of children in research however needs a genuine sharing of decisional power [19] and involves exploring children’s perspectives from children’s points of view and challenging conventional adult-led research processes [20].

The genuine involvement of children as co-researchers is useful for understanding children’s perspectives of the world, acknowledging that children know best about their own views and experiences [21]. The advantages also include obtaining easier access through peer groups, achieving genuine results by adopting the child’s perspective, and asking the right questions by prioritising the research agenda and posing the children’s research questions [17]. Those children taking part can themselves be empowered through their active involvement in research, which can also support personal development such as improving study skills and critical thinking and boosting self-esteem and confidence [17]. Children can be involved at different stages of the research process, with different levels of participation and different methods [21].

While ethnobiology has been at the forefront of the application and development of participatory methods [22], scientific papers explaining and discussing participatory research approaches in detail are rare. Instead, in usual publications in ethnobiology the methods chapters – participatory or not – are generally no longer than a few paragraphs and solely report on the methods applied, while mostly lacking information about the whole research processes including entry to the field site, building rapport with local people, reaching agreements on research questions or aims, guaranteeing benefits for local communities, abandoning field sites or feeding back results. These themes are likely to be seen by authors as a routine rather than core element of insight that might drive science ahead more than perfectly compiled result sections. The lack of detailed descriptions of research processes hampers a comprehensive evaluation of the research, including in ethical terms, and encompassing local challenges and needs addressed by the research or the benefits to local communities.

This paper presents and discusses the research processes and methods developed in the course of a three-year research project on wild plant gathering, the involvement of children as co-researchers and the project’s indications for local impact. In doing so, our objective is to encourage and intensify a discussion on research processes and methods in journals covering topics in the field of ethnobiology.

## Methods

### Research area

Research was conducted in the Grosses Walsertal, a sparsely populated mountain valley characterised by alpine farming in Vorarlberg, the westernmost province of Austria. Approximately 3400 people live on a surface area of 192 square kilometres in the six municipalities of Thüringerberg, St. Gerold, Blons, Sonntag, Fontanella and Raggal. There are approximately 180 agricultural operations, of which around 40 % are certified organic.

The Grosses Walsertal is characterised by a dispersed settlement pattern on its hillsides. Five of the six municipalities have a primary school, while the other municipality has two. The smallest school is in Marul (part of Raggal municipality) and at the time of the research had seven pupils aged between six and ten. The largest school in Thüringerberg, at the entrance to the valley, had 44 pupils in three classes. Additionally there is one secondary school in the community of Blons, but many pupils commute out of the valley to attend secondary school.

Since 2000 the valley has been designated a biosphere reserve [23]. Biosphere reserves are known for their rich biodiversity. In the Seville Strategy of 1996 a key direction is that the human dimensions of biosphere reserves should be reflected more fully [24]. The Großes Walsertal Biosphere Reserve is known for the strong link between local people and biodiversity, with gathered plant species being used as medicine for humans and animals, as food, for customs and ornamental purposes, and for cultivating home gardens, small arable plots for subsistence and orchards.

Due to the principle guidelines of the Grosses Walsertal Biosphere Reserve, the education curricula of local schools include ecological and social aims [25]. The sustainable use and processing of natural resources are important contents in education. A school for all senses should encourage children’s creative development. The children should be made aware of cultural values and natural resources as the main means of livelihood here. Good communication between generations as well as the appreciation of older, more experienced people in the valley should be fostered, but young people’s opinions and concerns also have to be acknowledged to enable their participation in the community [25].

### Research context

The research methodology outlined in this paper was developed in the context of the “Monitoring of Biocultural Diversity in the Biosphere Reserve Grosses Walsertal, Vorarlberg, Austria – The use and management of biodiversity of crops, cultivars and wild gathered plant species” project, funded under UNESCO’s Man and Biosphere Programme. The general idea for the project was created by the biosphere reserve management, who met with the authors in their quest for cooperation. Together we defined the aims for the project.

The principal aims were: 1) to document the diversity of wild plant species gathered by local people with state-of-the-art interdisciplinary methods, 2) to highlight the close link between biodiversity and local culture, and 3) to actively support various local initiatives concerning the sustainable conservation of biodiversity and biosphere management by involving these actors in the research process and disseminating the results.

### Research process and method

Research was divided into four phases between the years 2008 and 2010 (Table 1). In phase 1 freelist interviews and participant observation with local people was done by the first author of the paper. In phase 2 school workshops were held by the first author and children interviewed family members with structured questionnaires. In phase 3 children and researchers co-produced participatory videos. In phase 4 indications for the impact of the project were investigated.

**Table 1:** Overview of the research design in four research phases

Research phase	Main objectives	Methods	Samples	Outcomes and intended impact
1	Introduce the project to local people	Introduction of project in local newsletter, participation in plant-based and everyday life activities	Recipients of local newsletter, Alchemilla and Bergtee project members, daily encounters	Confidence and trust of biosphere reserve management and local people
	Investigate domain of wild gathered plant species	Freelisting Participant observation	Freelisting: snowball sample of 36 recommended wild plant experts, participant observation of plant gathering and processing with local people	Documentation of local knowledge, popular and scientific publications
2	Strengthen awareness about wild plant gathering	Preparatory and follow-up school workshops; two information letters to parents	14 classes of all primary schools in the valley	Improved information and awareness about wild plant gathering of children and local people
	Investigate intracultural knowledge variation and motivations	Structured questionnaires	Family members and friends of children, *n* = 506 people	Insights on knowledge variation and motivations, popular and scientific publications
	Encourage knowledge transmission	Children as interviewers	14 classes of all primary schools in the valley	Knowledge transmission through 189 children interviewing 506 respondents
3	Investigate children’s view on wild plant gathering	Two 5-day participatory video workshops with children interviewing local experts	All 17 children of St. Gerold primary school, 10 volunteering children from the valley, interviewing eleven local experts suggested by children	Children’s view on wild plant gathering, two participatory videos, 20 min each
	Encourage transmission and dissemination of local knowledge	Screening of participatory videos at local cultural festival; publishing of internet links to videos in local newsletter; DVDs to lend in local libraries	About 200 visitors of the screening, >500 views of each video in an online video channel,	Dissemination and transmission of local knowledge through participatory videos
	Empower children	Participatory video filmed with children with broad decision rights for children	All 17 children of St. Gerold primary school, and 10 volunteering children from the valley	Enhanced knowledge of plant gathering, skills in video production
4	Evaluate impact	Semi-structured questionnaires Participant observation	Questionnaires: ten children having taken part in the participatory video workshops and having used plants since then; participant observation during and after research phases 2–4	Indications about local impact of the research project

#### Phase 1: Freelist interviews and participant observation

The main objectives of phase 1 were to introduce local people to the project and for the scientists involved to become familiar with the local context by investigating the domain of wild gathered plant species. This phase took place between July and September 2008. Thirty-six local people (34 women, 2 men) were interviewed by the first author using *freelists* and subsequent semi-structured interviews [26, 27].

Participant observation of plant gathering and processing with local people deepened our understanding of the relevance of wild plant gathering in the *Grosses Walsertal* and accompanied the whole research process throughout all four phases [26]. The first author of the paper participated in plant gathering and processing activities with local people and took part in several activities within two local plant projects - the *Alchemilla* project (courses on soap making, balsam making, herbal retreat seminar) and the *Bergtee* project (annual meetings for all plant-gathering women, gathering plants and mixing tea blends with project leaders, packing and distributing the tea) (see [28] for more information on these projects).

The first author also built up a rapport with local people by taking part in everyday activities such as shopping, library events, sports activities, community festivities, cultural activities and attending church services. A publication in the local newsletter introduced the project to a wide range of local people [29].

#### Phase 2: School workshops and structured questionnaires

The main objectives of phase 2 were to enhance local people’s awareness of their wild plant gathering practices, to investigate intracultural knowledge variations and motivations for wild plant gathering and to encourage knowledge transmission.

We therefore approached the inhabitants *via* the valley’s seven primary schools in spring 2009. The first author of the paper initially informed the Grosses Walsertal committee for education and culture about the research plans and then discussed them in a formal committee meeting. The committee included, among others, the president of the committee, a headteacher, a project leader in the Bergtee project, the heads of local cultural communities and the manager of the biosphere reserve. She then contacted the heads of the primary schools to discuss the idea and later involved teachers and local actors in order to design the methods approach and organise data collection. The parents of all participating pupils were told about it in a letter outlining the project, including its partners, aims and anonymity of data. In two schools research plans were also presented in a parent-teacher conference and in two other schools parents were also informed by the headteachers themselves. The research was also announced in the biosphere reserve’s local newsletter.

Research was undertaken in all 14 primary school classes comprising a total of 189 pupils aged between six and ten. We started by running workshops on wild plant gathering for each class. The methods used in these workshops were based on long-term experience with children and school workshops, as well as the first author’s pedagogical education. The aim of the workshop was to arouse the children’s curiosity in the topic and increase their knowledge of wild plant gathering.

At the start of the workshop the children were asked if they were interested in plant gathering in general and whether they would be willing to support the research project as co-researchers.

**Fig. 1:**
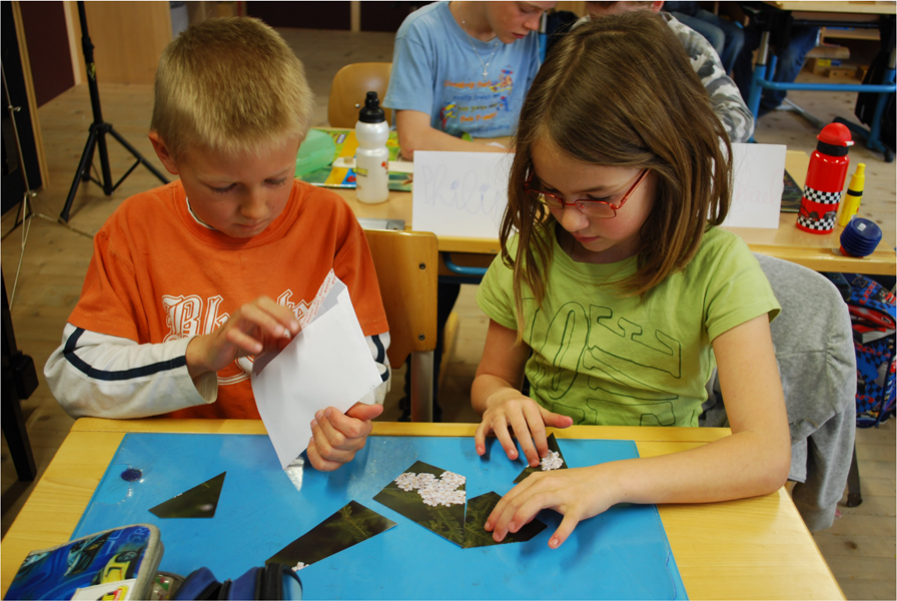
Pupils compiling plant puzzles. School workshop in Blons (Photo: Grasser 2009)

A poster was then prepared and the pupils were asked which plants came to mind that grow in the valley and have uses. We wrote down the children’s answers on the poster. Afterwards the pupils were asked to imagine that they were one of the plants that had been mentioned and to say what he or she is used for. The children stood in a circle and threw a ball from one child to another as they mentioned the plants. In a next step, the pupils were asked to put together 20 puzzles illustrating photographs of the 20 plant species mentioned most frequently in the freelists in phase 1 (Fig. 1). The pupils then presented the completed puzzles plant after plant to all their classmates, explaining which plant species can be seen and what they know about them. Dried and fresh plants as well as products made out of them were then presented. For example, pupils could try a balsam made from *Calendula officinalis,* touch and smell different herbs such as *Mentha sp.,* or taste dried berries of *Vaccinium myrtillus.*

At the end of these workshops we presented a closed-ended questionnaire consisting of five different sections on five pages and the children were asked to complete it with several family members separately as homework. In sections one to three, more detailed information about the 20 plant species mentioned most frequently in the freelists was queried. The 20 plant species were listed in rows in the first column of a table and respondents were asked to tick in the following columns of section one if they knew the respective plant species and used the plant species for eating, drinking, medicine, veterinary medicine, customs or other purposes. In section two, respondents were asked to tick if the leaves, flowers, fruits, seeds, roots, bark, shoots or resin were gathered. In section three respondents were asked to tick if they acquired the respective plant species themselves, acquired them from neighbours or friends in the area or bought them. If the respondents acquired the plants themselves, they were asked to state whether they grew them in their home garden, gathered them from meadows near their home, gathered them from the *Maisäß* (pastures in the lower mountain regions used before and after summer as the “middle station” to the Alp), the *Alp* (pastures in the higher mountain regions used during the summer months), in the forest or somewhere else. The sequence of the 20 plant species was distinct in the three sections. Multiple answers were possible.

In section four, motivations for gathering wild plant species were investigated using Likert scales. We proposed seventeen different motivations, which were created from statements given during the semi-structured interviews after the freelist interviews in Phase 1. The respondents were asked to mark in the questionnaire if the proposed motivations applied to their personal reasons for collecting wild plants (“1” signified very high and “5” very low agreement). In section five socio-demographic data about the respondents were investigated and free space for additional comments was provided.

The questionnaire was designed in a child-oriented way and included drawings and invitations for activities, such as looking outside to see which plant species are growing or checking which plant-based products could be found in cupboards in the house.

Sections one to three were checked for quality, practicability and comprehensibility in a pre-test with two children interviewing a parent or grandparent. Section four was pre-tested with nine adults during a meeting with the headteachers and in the committee for education and culture. Comments and suggestions from the test persons and schoolteachers were taken into account in the final version of the questionnaire.

Every pupil received four copies of the questionnaire. Children were prepared for the interview task by being shown the questionnaires and discussing each section. They were told what to ensure when interviewing family members, e.g. taking approximately half an hour for an interview, not being disturbed during the interview, explaining the questionnaire to the interview partner, asking everyone the same questions in the same order, filling in the answers in the correct columns *etc.*. In a short role play they tried out interviewing in pairs. General themes about science and research were not discussed due to time restrictions.

The teachers were asked to collect the questionnaires after they had been completed. The task was to bring all four questionnaires back, but this was not something we either forced them to do or monitored. At least one questionnaire from each child was requested.

The data from the questionnaires were entered into an MS Access database with the assistance of five women from the *Fontanella* community, who were paid a standard wage for the area.

In the school workshops, the teachers were involved in photographing the workshop and taking notes about the plants and plant uses mentioned.

The authors of this paper performed the data analysis. For the discussion of results and clarification of questions or ambiguities, the leader of the *Bergtee* project was consulted. Data were analysed descriptively first and results returned to the pupils in feedback workshops that took place about one month after the initial workshops. The children were introduced to scientific work in these workshops, and results were presented and discussed in a child-oriented way, including an emphasis on the importance and implications of such results.

At the start of the workshops, the children were asked to guess whether women or men and older or younger people knew more about wild plant species. They expressed their opinion by holding up different coloured cards for each answer. We reduced the presentation of the results to a minimum, which seemed a relevant way for us to communicate the main message to the children: to know who to ask when they wanted to know more.

In a second step we discussed the other results of the questionnaire with regard to how the interview partners use the plants, which plant parts are used and where the plants are gathered. For each section the pupils could fill in a kind of a crossword puzzle while we talked about these results so that they could take the results home.

In a third step we went out of the classroom and collected some of the wild plant species that had been mentioned. We then ended the workshop by tasting and enjoying the gathered wild plant species with the wild plant products we had brought along, while listening to a plant fairy tale.

Preliminary results were fed back to the survey respondents by the children who handed over an information letter and the crossword puzzle.

#### Phase 3: Participatory video workshops

The objectives of phase 3 were to investigate the children’s views on wild plant gathering and encourage the transmission and dissemination of local knowledge to a wide range of people.

As expressed by teachers and parents orally, the school workshops and questionnaires were positively received by local people, and the primary school teachers requested a continuation of our work on wild plant gathering in schools. In order to achieve the project objectives of encouraging knowledge transmission about wild plant gathering and disseminating local wild plant knowledge to a wide range of people, we proposed working with participatory videos on wild plant gathering with the children.

We presented and discussed the idea with the biosphere reserve manager, the committee for education and culture and the headteacher of one primary school.

The first participatory video workshop was held during regular school time in the primary school in *St. Gerold* (all 17 pupils aged between six and ten participated) in May 2010, at the headteacher’s request. A second participatory video workshop was announced in the biosphere reserve’s local newsletter and ten children (aged between eight and thirteen) registered from the communities of *Thüringerberg*, *Blons*, *Sonntag*, *Fontanella* and *Raggal*. The second workshop was held free of charge during the children’s summer holidays in July 2010.

The five-day workshops were run by the first author in cooperation with a professional filmmaker who had prior experience of making participatory videos with children. The workshops followed a defined sequence (Table 2). Again, the parents gave prior informed consent by signing an information letter and indicating their willingness for their children to participate in the video workshop. The gatherers featured in the video also stated in writing that they were willing to participate and that the video could be released afterwards.

**Table 2:** Process and responsibilities for producing the two participatory videos

	Day	Objective	Activity	Children’s responsibilities	Workshop leaders’ responsibilities
Introduction to video-making	Day 1: ½ day	Familiarisation of children with making videos; introduction to roles of video team	Teaching and experimenting with video material and camera equipment	Learning about making videos	Teaching about making videos
Writing of storyboard	Day 1: ¼ day	Composition of storyboard	Collect children’s knowledge about wild plant gathering and use; write short stories about especially knowledgeable gatherers and their practices	Generating ideas, writing of storyboard, drafting of interview questions, decision on storyboard	Introducing storyboard writing, decision on story board, defining topic of wild plant gathering
Organisation of video recording	Day 1: ¼ day	Preparation of participatory video recording	Proposing and contacting wild plant gatherers and arranging filming on site	Proposing gatherers to be interviewed Asking gatherers for their availability if he/she was a relative	Contacting proposed gatherers and arranging filming on site
Video recording	Days 2–4: 3 days	Recording of raw material for videos	Distribution of roles to storyboard team and backstage team, taping of videos	Taping of videos	Guiding and supervising the taping of videos
Intermediary raw editing	Days 2–4: 3 days	Gaining raw versions of the videos to discuss strengths and weaknesses every morning, introducing children to editing process	Raw editing of film material	None	Full responsibility
Development of a frame story	Day 5: ½ day	Writing the storyboard for the frame story to embed videos in a larger context	Brainstorming for storyboard, writing stories and decision for selected story	Bringing costumes for studio recording; suggesting title	Guiding and supervising the development of the frame story
Recording of frame story	Day 5: ½ day	Recording of frame story	Recording frame story, making and recording music to be included in the video, designing of cover of DVD box	Recording the frame story, making and recording music, drawing a cover	Guiding and supervising the recording of the frame story
Discussion of raw version of complete video	Day 9: ½ day	Adjusting ideas of children and gatherers with ideas of workshop leaders	Presentation of videos to children and all involved gatherers starring in the video, discussion of changes	Suggestions for changes	Presentation of videos; taking up suggestions for changes
Final editing	Day 14	Producing final video	Final editing following comments received	None	Full responsibility

At the start of the workshop the children were asked if they remembered the plant project from last year. They could remember a lot of the activities and stated that they were very interested in being a co-researcher again. They were curious about making a video and committed to participating in the workshop.

**Fig. 2:**
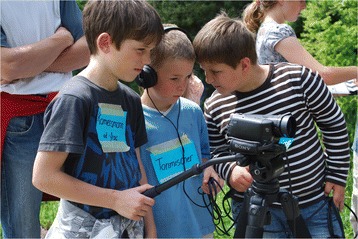
Training with the video equipment. Introducing the workshop in the St. Gerold primary school (Photo: Grasser 2009)

During a brief introduction, the children were introduced to video-making. They learned how a film is developed, what roles (producer, camera operator, sound engineer, interviewer, storyteller, actor etc.) are necessary and how to use a video camera (Fig. 2).

The different duties were then practised with short games and exercises. In a next step we focused on the plant topic, which the children already associated with the first author who was now the leader of the participatory video workshop. The children were asked to identify interesting themes related to plant gathering and use and to write short stories about local people who are knowledgeable in wild plant practices and their wild plant-related activities in the community. After that, the children and the first author and filmmaker decided together what the most exciting topics were and which stories it would be possible to film according to the vegetation in late May. In five groups the children drew storyboards and devised interview questions. The following days were organised by the workshop leader in arrangement with knowledgeable gatherers who the children proposed should be in the videos. Weather conditions, such as heavy rainfall, plants growing at that time and the gatherers’ availability, presented a major challenge day by day.

Within three days, five stories with five gatherers were filmed in five different places. During the video production, each child had a defined role and duty to fulfil. We arranged two teams – one responsible for the main film and the second for filming the children/film-makers while they were making the main film – in order to record the development of the main films, again from the children’s perspective. After each school day, the workshop leaders undertook a first raw editing of the film material to discuss strengths and weaknesses with the children the following morning. Each day we changed all the children’s roles so that everyone had an opportunity to fulfil each duty.

On the fifth day a frame story bringing together all the clips along a common theme was written. Children were asked to bring costumes for the studio recording and come up with suggestions for a film title. This frame story was also filmed on the fifth day and a cover of the DVD box with contributions from each child was designed. The filmmaker gave the children insight into how editing works and they were to try this out for themselves on short sections.

We then finalised the whole video and presented the results to the children and all the experts involved in a workshop to discuss any requests for changes. Immediately after this workshop the children were asked for feedback.

#### Phase 4: Impact evaluation

One year after the participatory video project, in August 2011, the first author called all the headteachers to informally inquire about what impact the project had had locally. They were then asked to recommend interview partners among the pupils. The selection criteria for the children were those who said that they had been using plants since the plant project, the children who had taken part in the video project, and children who were available for interview. Ten children were interviewed using a semi-structured questionnaire about their memories and the lessons they had learned. Participant observation by the first author, who lived in the valley for the time of research, essentially contributed to the evaluation of the project’s impact. Participant observation included playing with the neighbourhood children, accompanying them on their way to school or leisure activities, taking part on family trips, spending time on alpine pastures with farmers and their children as well as everyday activities with local people, as explained for phase 1.

Despite such measures to evaluate impact, we admit that they were not comprehensive enough to provide evidence about the impact of the project. Rather than that they gave us indications about which impact the research project had. Future research is asked to test these indications and to support or refuse our findings through evidence.

### Ethical considerations for research with children

We committed ourselves to observing the International Charter for Ethical Research Involving Children [30]. The seven key commitments of the Charter are listed below for a discussion of the study’s ethical conduct in relation to the involvement of children. This section has only been slightly modified since it was first published in [31].

In planning the research process and in all our interactions with the children we followed commitment 1 “Ethics in research involving children is everyone’s responsibility” and commitment 2 “Respecting the dignity of children is core to ethical research”.

We followed commitment 3 “Research involving children must be just and equitable” by ensuring that all project-related tasks were co-designed with teachers, adapted to the pupils’ knowledge level and included in the school routine, and that participation was voluntary. It was ensured that the project-related tasks did not involve additional work for the pupils: when the children were given the questionnaire task, they were not given any other homework by the school. The timeframe of the video workshop was during regular school time. Participation in the video workshop during the summer holidays was voluntary.

We followed commitment 4 “Ethical research benefits children” by maximising the learning experience for the pupils. The first author organised wild plant workshops with the pupils before and after data collection to give them information about the topic and then feed back the results. The questionnaire was designed in a child-oriented way and provided opportunities to learn about wild plants. In the participatory video project, the children learned about plants and about video-making, but there was also social interaction as they had to cooperate in teams and interact with the plant experts. All the children had the opportunity to try all the tasks involved in video production. Some of the results from the study were published in the biosphere reserve’s local newsletter, which benefited the children by raising the profile and boosting the value of their work.

We followed commitment 5 “Children should never be harmed by their participation in research” by being attentive during all interactions with the pupils and avoiding any potential risks of harm when planning the study. In particular we ensured that the workload for pupils remained balanced with leisure activities and no pressure was placed on them to complete the assignments. During the outdoor video work, safety was ensured by taking appropriate alpine paths and carrying a first aid kit.

We followed commitment 6 “Research must always obtain children’s informed and ongoing consent”. For phase 2, we obtained prior informed consent from the biosphere reserve committee and the headteachers and ensured the commitment of the committee for education and culture and the parents of the children involved. The project activities were also pre-announced in the local newsletter which is sent to every household in the valley. Informed assent was sought from the pupils during the first workshops. The children were then told about the study and given the opportunity not to take part. However, we are aware that in school settings children may easily feel obliged to co-operate [32]. We did not receive any objections to them participating in the research activity. For phase 3, informed consent was received from the headteachers, teachers and parents of the pupils as well as from the gatherers who were filmed and gave their written consent for the videos to be published.

We followed commitment 7 “Ethical research requires ongoing reflection” in all interactions with the children by reflecting on our practices and values and the influence these had on the pupils.

## Results and discussion

### Knowledge and use of wild plants (phase 1)

Each respondent mentioned on average 25 wild plant species and their uses and knowledge about the use of 140 different plant species was documented. Most plant uses were mentioned for medicine and food. Two local wild plant projects (*Alchemilla* and *Bergtee*) play a vital role for promoting and value wild plant gathering [28]. The results of this phase were published in popular [33–35] and scientific [28] publications.

### Knowledge variation and motivations for wild plant gathering (phase 2)

“Women, older informants and homegardeners report more human medicinal applications and applications in drinks than men, younger informants and non-homegardeners; farmers know a greater variety of veterinary medicinal applications than non-farmers; the place of residence relates significantly to food and veterinary uses” [36]. Four main motivations of gatherers were identified: most inhabitants gather because of the highly esteemed product quality of wild plants and the fun of gathering, far less because of tradition and only a few for generating income [31]. The results of this phase were published in popular [33, 35, 37] and scientific publications [31, 36].

### Children’s view on wild plant gathering and plant uses (phase 3)

**Fig. 3:**
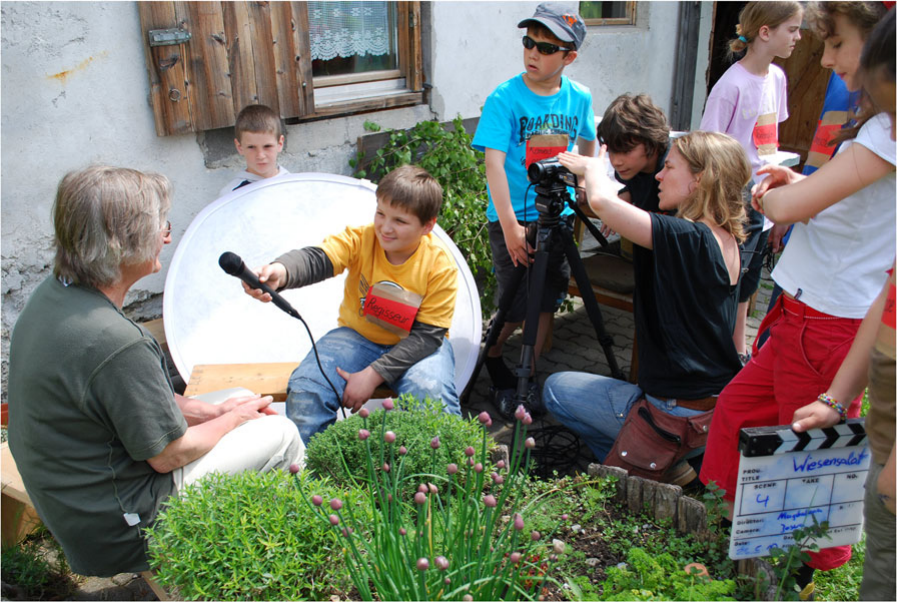
Pupils interviewing local plant experts in their community. St. Gerold primary school (Photo: Grasser 2010)
- (2) “Ein Zwerg kaut am Berg Kraut”, a dwarf story in which nine little dwarfs searched for a remedy to heal their sick king. They went to experienced adults in the valley to ask for help. With each expert the dwarfs prepared another plant-based remedy. Finally the root of masterwort healed the king (Fig. 4). The name of the video literally means “A dwarf chewing herbs in the mountains”

The videos produced give insights on childrens’ perceptions of wild plant gathering and adult’s expertise about wild plant gathering. The frame stories elaborated by the children were:

- (1) “Kraut im Bild”, a newscast in which six children moderators invited twelve children researchers as guests (always two at a time in six sections), who were briefly interviewed about their plant expertise. Together the moderators and researchers then went out of the studio into the field and visited local adults who were experts in collecting wild plants, conducting interviews and making different plant products with them (Fig. 3). The name “Kraut im Bild” is linked to the prime time news programme on Austrian TV, also known by all the children, called “Zeit im Bild”, *i.e.* communicating that herbs (“Kraut”) are being featured by the news channel.

**Fig. 4:**
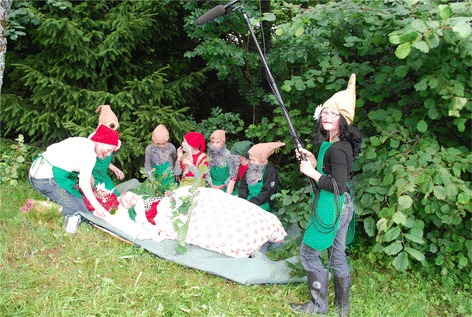
Filming of the frame story. Summer workshop in the Grosses Walsertal Biosphere Reserve (Photo: Grasser 2010)

Children asked detailed questions when interviewing gatherers and tried to get information and understand processes involved in wild plant gathering. E.g. they asked exactly where and how to gather plants, how to use plants (e.g. making ointment, fumigate with roots) and what a plant or a special remedy is used for and how to use it. Several children also wanted to know from whom their interview partners have learned their plant knowledge. Children also asked what the interview partners like about gathering wild plants, making ointment or other herbal remedies and why they do it. They asked for special experiences connected to plants - also in the gatherer’s childhood - and therefore inquired stories besides plant-based information. One sequence of the video was most exciting for the children as they commented themselves: digging Masterwort (*Peucedaneum osthrutium*) in an Alpine pasture and set it on fire for fumigation in a dark old stable was a real adventure.

The videos were first screened at the *Walserherbst* cultural festival in September 2010, six weeks after their production. All the children and local experts who had participated in the video workshops received an invitation with free admission to the festival. DVDs were given to all the participating pupils and experts for their personal use and to all six local libraries. The videos were also published online [38, 39].

In addition to the videos, the results of this phase were published in popular publications [35, 37, 40].

### Local impact of children’s participation (phase 4)

The local impact of the research was not published before and is thus presented in more detail below. We adapted a framework originally designed for evaluating the impact of childrens participation in development projects to discuss indications for local impact of the research processes and methods. The original framework builds on five areas of potential impact, which are personal, familial, communal, institutional and negative, each including several subcategories [41]. We adapted the extensive subcategories of the framework to the context of our project and only report on those relevant for our context.

### Personal impact

#### Self-confidence

An increase in self-confidence is a commonly mentioned result of participatory activities with children [41, 42]. In this research project, the children learned to collect data through questionnaires in phase 2, and co-produced videos in phase 3. They became confident of being able to produce something valuable for a general and also an adult audience, as confirmed by the headmaster of the primary school St. Gerold. Parents of the participating children declared that the dissemination of results in local newsletters and at the local film festival has given the participating children pride in their knowledge and skills, which in turn boosted their self-confidence. The children received a very positive reception and their work was highly acclaimed both within the valley and elsewhere.

The herbal experts who were interviewed in the video were also proud to see themselves on the big screen at the cultural festival, as local people from the audience of the *Walserherbst* cultural festival noticed. Awareness of the value of people’s own knowledge is one of the results of the participatory video project, in which not just the product but the whole process counts [43].

#### Knowledge and skills

Participatory research in general, as well as with children, requires the provision of some kind of training before the research can begin [19]. We used the school workshops for this purpose where the topic of wild plant species and the questionnaire were introduced and directions were given as to how to complete the questionnaires. Although capacity building should not only introduce research methods but also basic ideas about research to empower children [19], we were unable to meet this objective sufficiently.

Children’s awareness and knowledge of the research topic, the gathering and use of wild plants, was increased and many were motivated to gather and use plants themselves, as far as we could observe. For example, a mother reported that she had observed her daughter playing with her little brother in the meadows. He had hurt himself and had a small cut on his finger. The girl picked ribwort (*Plantago lanceolata*) and tied the leaf around her brother’s finger. The mother heard her telling him that she had learned at school that this would stop his finger bleeding.

Children acquired skills in plant gathering and processing by performing these tasks with the interviewed plant experts. Some told us later about which plants they gathered themselves, *e.g.* for making tea or preparing an herbal spread.

Children also acquired practical skills in conducting interviews and making films. They performed all the roles involved in a small professional film production team and had the opportunity to try out all the tasks involved. The children all seemed to have their own particular strengths (technical, organisational, storytelling, conducting interviews, role-play *etc*.). Whereas the development of skills is a predictable impact of the involvement of children as co-researchers, the actual success in skill development is linked to the methods applied and needs to be evaluated [42].

Videos do not replace direct interaction with plants. Touching, smelling and tasting the plants is essential for a holistic approach to learning and learning using all the senses. Our approach was to capture children’s attention and motivate them to participate through video recordings, the backstage team and the story. This approach meant that children who did not have much interest in plants had learned something about the plants, along with video recording. Others were more interested in the plants, plant gathering, plant processing and plant products. The project offered each of the children something to capture their interest.

#### Personal development and social development

Children highlighted that during the video project they learned about the importance of teamwork. They experienced being involved in a project and creating something worthwhile, which some intended to carry on after the project. For example the leader of the *Bergtee* association reported that a couple of months after the project that a ten-year-old girl, who had participated in the video workshop, had come to her house and asked if she could join the *Bergtee* association. Through the video she had learned that it was open to everyone and that the more women there are collecting herbs the better. So this girl joined the *Bergtee* association and gathers herbs with its other members. Subsequently, two other girls from the participatory video workshops joined the *Bergtee* association too. Working in a team with peers and joining associations both is suggested to make an essential contribution to the development of children’s personalities and social competence [41].

#### Positive channel for energy and creativity

During the video workshops the children’s enthusiasm was evident. They worked hard and were willing to continue even on a public holiday. The teachers expressed their surprise at some pupils who had fairly weak performances at school but who impressed them with their great performance during the video workshop. The children were given the possibility of trying out different roles, which allowed them to identify their individual strengths and weaknesses and develop their creative potential as the partaking headmaster of the primary school St. Gerold suggested.

### Familial impact

On a family level, the exchange and transmission of knowledge was intended to be fostered by the project and - as several parents of participating children mentioned - the status of children was enhanced within families since children now became researchers, video producers and experts on wild plants. A mother reported that her daughter came home with a bunch of dandelions (*Taraxacum officinale*). The girl wanted to make syrup of dandelion (*Löwenzahnhonig)*, but neither knew how to prepare it. However through the school workshops the daughter knew that older women would be likely to know about wild plant uses, so they went to their neighbour and asked her elderly aunt.

**Fig. 5:**
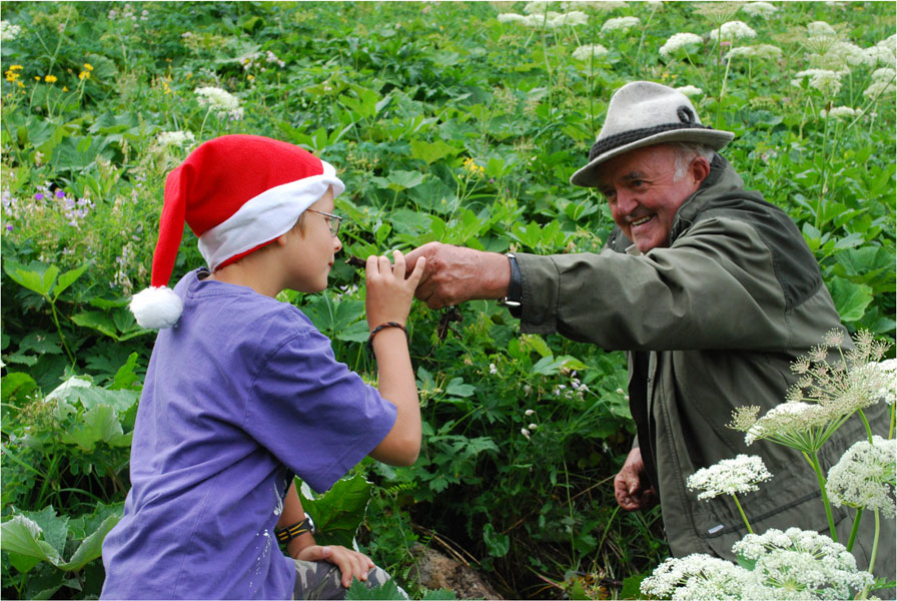
Knowledge transmission: A local farmer shows the root of Masterwort. Participatory video workshop in the Grosses Walsertal Biosphere Reserve (Photo: Grasser 2010)

A mother reported that her son brought her a piece of masterwort (*Peucedanum osthrutium*). She just had come home from having a tooth operation and had significant pain in her teeth. Her son gave her the root and said this would help with toothache. The mother was a little suspicious, but then together they watched the video with the sequence of masterwort and the local experts’ explanation (Fig. 5).

### Communal impact

Through the participatory research project, wild plants became a hot topic in the valley. The community was regularly informed about project activities through local newsletters and informal dissemination of information. We received many reports of how exchanging knowledge and recipes for wild plant products had been encouraged by the project.

For example, we observed women talking about wild plant uses and becoming aware that they could prepare products themselves, such as syrup made from the flowers of the elder tree (*Sambucus nigra*). They were aware of this product from their childhood but had never prepared it. Through the children’s activities and interest, the parents gained motivation for wild plant gathering and use. One elderly woman who collected herbs for the *Bergtee* association decided to stop her contribution one year. However the next year she brought a bag of dried herbs to the *Bergtee* leader again and explained that her grandson (a boy who was involved in the video workshops through the primary school in *St. Gerold*) had become interested in plant gathering so she had to continue to teach him about the plants that can be gathered, plant uses, gathering locations *etc*.

The focus on the topic of wild plant gathering culminated in the screening of the videos during the *Walserherbst* cultural festival alongside prominent films by renowned international filmmakers. Both videos were enthusiastically received by the audience, with local people apparently realising the value of their own local knowledge about plant gathering and use, which is suggested to strengthen local people’s identity (similar to [43]). Additionally, by watching the videos together, protagonists and local people were given the opportunity to discuss and reflect on the practices shown, and the participatory video thereby served as media for empowerment [44].

**Fig. 6:**
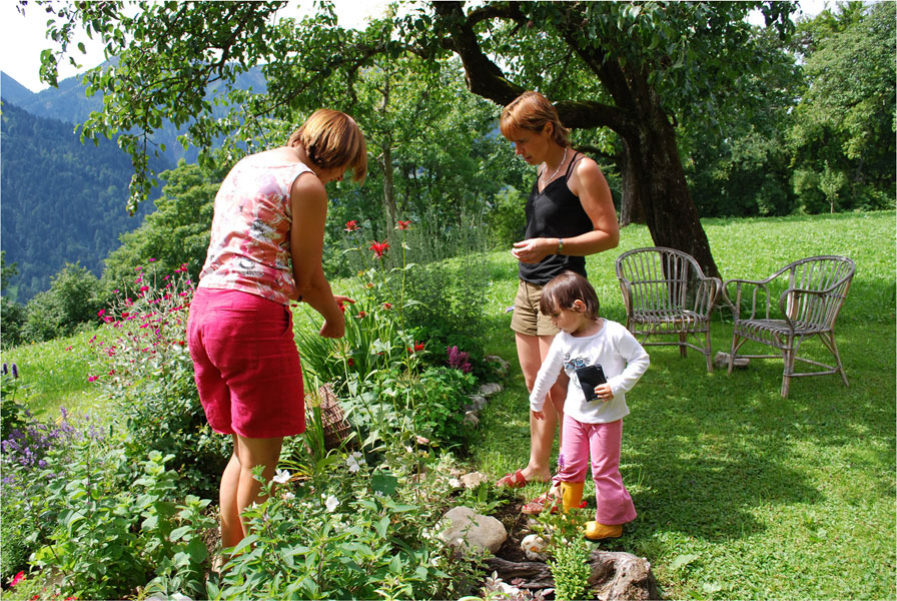
Neighbours talk about herbs and their use in and around the garden. Farmer’s garden in Blons (Photo: Grasser 2008)

Intergenerational transmission of local knowledge is essential for the vitality of local knowledge [45]. The school and participatory video workshops initiated a process of transmission of knowledge (Fig. 6), initiated through children’s activities. When the children asked older people about plant gathering and filmed and screened the videos, the adults themselves affirmed that they became aware that their knowledge is not that common and is not nothing special, as they often mentioned during the first research phases, but is important and worth being transmitted. Awareness of the value of people’s plant-related knowledge very likely could be raised. Children were observed by the first author as well as parents and grandparents and other local people (e.g. the leader of the *Bergtee* association) to become curious about plant gathering outside the project and to perceive their surrounding nature more keenly and to start to ask questions. The results of phases 2 and 3 were disseminated to and through the children, which again continued the discussion.

There are three common paths for transmitting cultural traits: vertical, horizontal and oblique ([46] in [47]). In the project we made use of all the paths: children interviewed their parents and grandparents (vertical transmission); parents exchanged their knowledge and children exchanged what they had learned (horizontal); and children without experts on plants at home (e.g. knowledgeable grandparents or parents) learned from other older members of the community (oblique transmission), especially in the participatory video project. Children also brought their knowledge (which they learned from other older people in the community) back home, which may be seen as a fourth path of knowledge transmission (from child to parent).

### Institutional impact

Although we did not intend to introduce ethnobotany and local knowledge into *formal* school-curricula, local teachers were inspired by the project activities to take local plant knowledge into account in their teaching. For example in the school that hosted the first video workshop, the teachers now include the videos as didactic material in the school curriculum.

The inclusion of local knowledge systems in formal school curricula has the potential to maintain or revitalise local knowledge in the communities [48]. However, it requires culturally representative ways of teaching. Therefore we did not teach wild plant uses in the primary schools, but let the local people talk and present their practices themselves in the questionnaires and participatory videos. An example from the Tsimane educational system in Bolivia shows that contextualised learning helps maintain local knowledge systems [12]. Here the children are taught about local knowledge in the Tsimane language by Tsimane teachers using educational materials in Tsimane. In our research we placed the emphasis on contextualised learning with our approach to let children interview their parents and grandparents, and by working with participatory video, where the children filmed local experts on plant gathering and use. By including local knowledge in formal education systems, intergenerational transmission may increase and it might be an important way of increasing pupils’ awareness and participation in environmental issues [48].

One year after the video production, the school hosting one video workshop won first prize for projects in Austria’s national “*Umweltzeichen*” competition, an ecological label for schools, for their video “*Kraut im Bild*”. This raised the school’s profile considerably.

Besides the local primary schools, the institutionalised herbal association *Bergtee* profited from the participatory video project: by being interviewed in one of the videos, the leader of the *Bergtee* association was able to advertise her *Bergtee* project to a broader public. The association acquired three more members after the screening of the videos: young girls who participated in the video workshop and are now gathering herbs for *Bergtee* with older women in the association.

### Negative impact

The family environment in particular has the potential to be negatively impacted by participatory projects with children. This may include conflicts of interest between parents and children, problems between siblings when only one child participates, disruption of power relations within families or communities, disappointments when projects do not turn out as intended or overburdening the children [41]. Although we were attentive to such instances, as also promoted by the International Charter for Ethical Research Involving Children [30], no negative impacts were reported within the scope of this project neither by parents, teachers nor the children themselves.

### Reflecting upon children’s participation

Children can be involved at various phases of the research process, to various degrees, in various forms of participation and with various methods (Alderson, 2001). In this chapter we draw on these factors to discuss the participation of children in the research project.

### Phases and degrees for participation of children

**Fig. 7:**
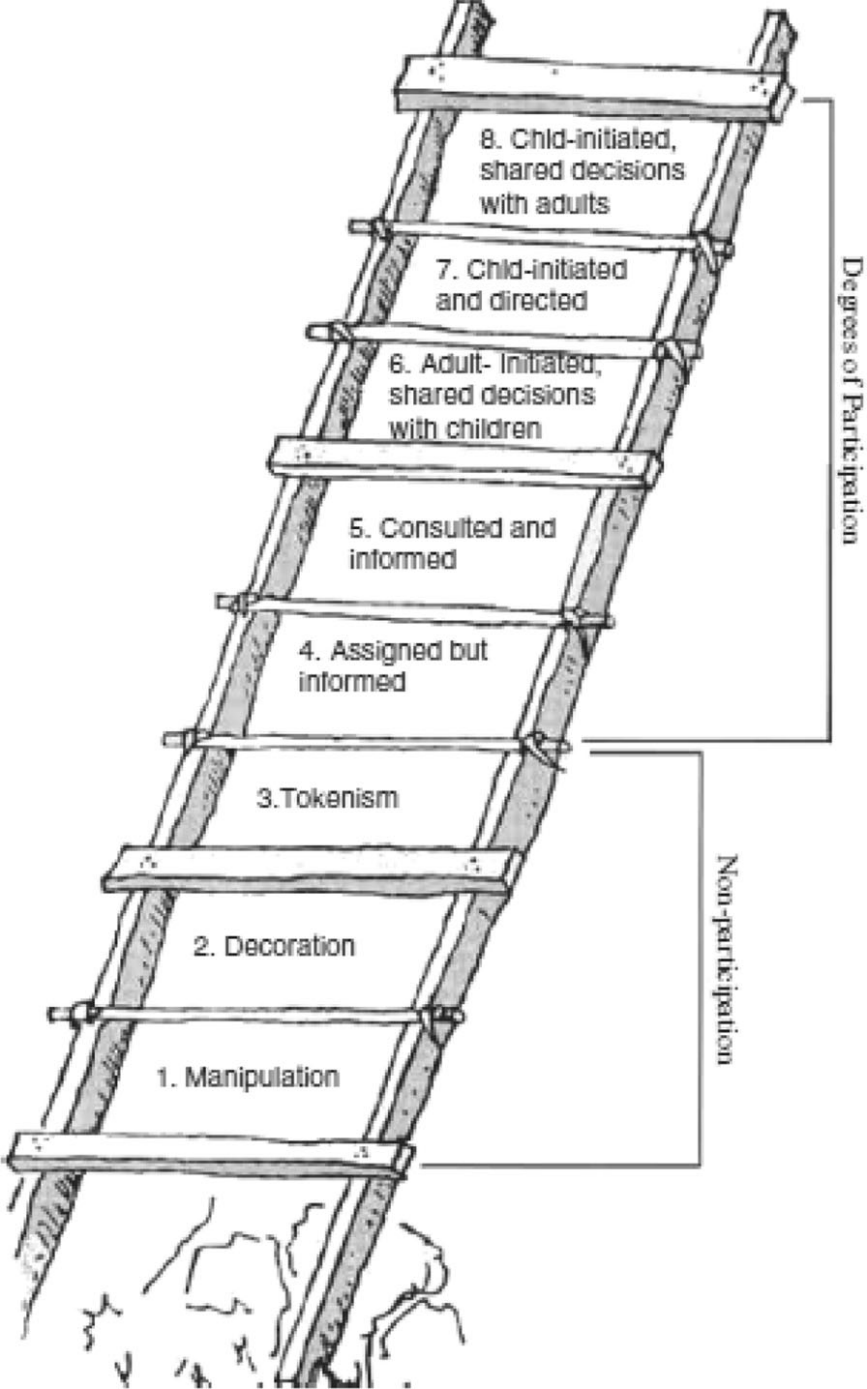
Ladder of participation to illustrate children’s degree of involvement (Source: [41])

The ladder model is one widely used example for reflecting on the extent of and form of children’s participation in participatory research projects [49]. It is comprised of eight rungs, ranging from three types of non-participation to five degrees of participation (Fig. 7). The types of non-participation are manipulation, decoration and tokenism. The degrees of participation are assigned but informed, consulted and informed, adult-initiated shared decision with children, child-initiated and directed, and child-initiated shared decisions with adults. The ladder model has also been criticised, however, for example for reflecting the participation of children who are only in the project and not the general societal context, for being culturally biased, for being used as an evaluation tool rather than a scheme for reflection or for implicitly stating that the higher up the ladder, thus the more the participation, the better it is and that a development from lower to higher ranks is being targeted [50].

Following this model, children were assigned but informed in research phase 2. They were involved in the data collection process as interviewers, but the research process and method was selected and designed by the researchers and supported by teachers. Children were introduced to the topic and informed about the results, but were not offered the opportunity to make decisions about the process. Although this kind of participation is the first degree of participation in the ladder model, it was not genuinely participatory when sincere power-sharing with children is identified as a pre-requisite for participatory research [17, 19]. Phase 2 might also not be called genuinely participatory since in the regular school setting children may easily feel obliged to co-operate [32] and participation might therefore not have been fully voluntary, another pre-requisite for participatory research [49, 50]. The other pre-requisites were fulfilled since in the workshops we ensured that the children understood the intentions and reasons for their involvement, and that they had a meaningful, and not merely decorative, role in the project [49].

Research phase 2 might thus lie on the edge of what we call participatory research with children. It suited its purposes of strengthening awareness about wild plant gathering among children and local people, encouraging knowledge transmission and data collection. The phase was valuable for ensuring the project objectives were achieved and involved benefits for local people and children [50].

Following the ladder model, phase 3 was adult-initiated shared decisions with children and most authors would agree that this phase was participatory since decision-making was shared. The first author came up with the idea of working on participatory videos and this was then presented and discussed with the key actors in the valley. The sequence for producing the videos and the topic of wild plant gathering was also predetermined by the researchers. Within these conditions, the children and researchers co-determined the content and storyboard of the videos, the local people to be interviewed and the scenes and point-of-view shots. The editing of the videos was again undertaken by the researchers.

Overall, the children were increasingly involved in the research project as it progressed. While in phase 1 children were not involved at all, children collected data in phase 2 and were involved in almost all the stages in phase 3. Phase 1 was essential for laying the groundwork. It was used to establish a rapport and obtain the confidence and trust of biosphere reserve management and local people in the project. This again increased local people’s willingness and motivation to participate in project activities. It was essential for the success of the project that parents, collaborators, key actors and ultimately the whole community also supported the project. Potential conflicts of interests between children, parents, teachers and other actors were thereby mitigated [41].

The involvement of children in the data collection in phase 2 presented a first entry point for work with the children. Phase 2 was positively received by local people and the teachers of the primary schools requested that our work on wild plant gathering be continued in the schools. This was only possible because the funding agency gave us relative freedom to undertake the research phases as long as the objectives were achieved. In phase 3 participatory videos with the children allowed us to continue the work with children and involve them in more stages of the research process while still meeting the project objectives.

### Methods for children’s participation

Children were offered a diversity of methods for expressing their opinions and experiences in participatory research approaches over the last two decades. Such methods include written, oral and visual ones and range from questionnaires or interviewing to drawing, diaries, mapping, ranking, transect walks, acting, theatre and many more [51–53].

In this chapter we discuss the main methods applied in this project with children: questionnaires and participatory videos.

#### Questionnaires

Quantitative methods such as questionnaires have recently been less favoured in research with children [54, 55] since they, with their stringent structure and focus on objectivity, were not perceived as children-friendly by researchers [54]. However, these experiences were challenged and it was highlighted that some children preferably engage in structured methods and do not fit into the assumption that all children prefer to draw or play [56, 57]. Reasons might include that the formality of questionnaires supports the children’s impression that they are doing serious research [57]. Children were thus found to be capable for responding to a diversity of methods and the most apt way for doing research with children is to recognize the diversity of interests and affinities of children and providing choices through participatory approaches rather than assuming an uniformity of interests of children [56]. In this project, questionnaires were preceded by school workshops where posters, games and puzzles were used as icebreakers [53] and alternating sedentary and moving activities were used. This was followed by the introduction of the questionnaire and the relating homework. We thus intended to provide a diversity of methods for supporting multiple of children’s interests and for making the workshops enjoyable (similar to [57]). However we admit that children were not given real choice of participation in the activities.

We argued that our questionnaires were designed in a child-oriented way with drawings and intermediary activities, as explained above and in earlier publications [28, 31, 36]. Terms such as child-orientation or child-friendliness might however be used as patronising terms and all research subjects, not only children, benefit from research methods which are adapted to their interests, backgrounds as well as local environments [58]. Also, the use of child-friendly methods may hide the benefits for the researcher and may reinforce the researchers’ assumptions about childhood rather than providing space for choice and development of children. A distinction between adult-suited and child-suited methods may thus be misleading and hiding the heterogeneity among children, their interests and experiences [59]. Alternative and better suited terms might be research-friendly or person-friendly research or research-participant-centred rather than child-centred research [58]. Considering such arguments, we believe that our questionnaire was indeed researcher centred as well as research-participant-centred: it fulfilled its purposes of providing entertaining data collection for the children, supporting knowledge transmission from research subjects to children and gathering scientific data.

#### Participatory videos

Participatory video is characterized by heterogeneity of approaches [60], and its use by far not only in research but also in applied projects to empower civil society [44]. Key characteristics of participatory videos with children are 1) the complex process of elaborating participatory videos, 2) the balanced focus on both the process of elaboration and the resulting output, and 3) linked to complexity and process orientation, the importance of the multiple relationships between researchers, children and community members [61].

1. In our case, we intended to reduce complexity through pursuing a defined work plan and at the same time giving space for flexibility and common learning, e.g. through possibilities for evaluation and feedback [62]. The sequence of the workshop and the topic were defined by the workshop organizers, whereas the content and the choice of interview partners was elaborated independently by the children. Whereas the work plan was not discussible, space for discussion and evaluation was reserved on three mornings when footage was reviewed as well as during the presentation of the videos four days after the school workshop.
2. Knowledge production during the process of the development and the final product are the aims of participatory video [63]. Production and transmission of knowledge on wild plant gathering was realized during video development when children were interviewing gatherers and through the public screening and availability of the final product. Furthermore the children developed personal knowledge and skills during the development process, as indicated above.
3. Multiple relationships around wild plant gathering were fostered during and after the participatory video project. The relationship between the first author, the teachers and the partaking children was already established through the school workshops and the first author was well known for the topic of wild plant gathering at the time of the video workshops. These established relationships and trust provided the basis for a smooth start of the video workshops. The recording of participatory videos then fostered the relationships between local gatherers and children. Local gatherers felt free and easy in interacting with the children and sharing their knowledge. They told their stories not to the camera but to the children and interacting with children helped the gatherers to be relaxed while being recorded.

Although videos are used since decades in research, they still lack recognition as academic outputs and are rarely presented as such by researchers [63]. This is also true for participatory videos in ethnobiology and ethnobotany and literally no scientific publications describing, discussing or analyzing in detail the process of development or resulting participatory videos are to be found in ethnobotanical journals. Rather than scientific outputs, ethnobotanical videos were intended as educational ethnobotanical videos for ethnobotany teachers and learners, ethnographic videos for broader audiences and local ethnobotanical videos for community members [64]. Such lack of scientific outputs from videos was suggested to be the case because visual accounts of cultural practices hardly fit to the text-based academic institutional practices [63], but also because of funders’ reluctance of supporting process oriented research [4, 61]. This lack provides also potential for further developments in ethnobiology.

## Conclusions

We have presented and discussed the research processes and methods developed in an ethnobiological research project in which children participated as co-researchers.

Children participated in various ways in the research process and the scientific output and local impact of the project was linked to the phases, degrees and methods of children’s involvement. Children’s involvement ranged from non-participation in phase 1 to punctual involvement as interviewers in phase 2, as co-researchers in participatory video making in phase 3, and again to non-participation in phase 4. Indications for local impact were found on personal, familial, communal and institutional levels and were most pronounced when children actively participated in the research process (phase two and three). Scientific output was generated from non-participatory research processes (phase one and four) as well as through the participation of children as researchers (phase two). The example of this research project thus shows how participatory involvement of children in ethnobiological projects can combine both, local impact and scientific output.

Participatory video (phase three) is known as an important tool for creating local impact in ethnobiology. However, less attention was put on creating scientific outputs from participatory video and e.g. the detailed methodological discussion of participatory video-making partnerships, impact evaluation or the presentation of videos themselves as scientific outputs bear potential in ethnobiology.

Research projects are frequently complex endeavours and time schedules are tight. However, if ethnobotanists and ethnobiologists are truly concerned with the impact and benefits of research processes and results for local communities, this research needs to be deliberately planned and evaluated and then reported and discussed in academic publications. We would appreciate any feedback on our methodological procedure in scientific publications, via e-mail or posts on ResearchGate [65].
